# Prevalence and Antimicrobial Resistance of Salmonella in Poultry Products in Central Ethiopia

**DOI:** 10.1155/2022/8625636

**Published:** 2022-12-20

**Authors:** Isayas Asefa Kebede, Tefari Duga

**Affiliations:** School of Veterinary Medicine, Wolaita Sodo University, P.O. Box 138, Wolaita Sodo, Ethiopia

## Abstract

Salmonellosis is a bacterial infection caused by salmonella, a member of the Enterobacteriaceae family. From December 2021 to May 2021, a cross-sectional study was carried out to isolate Salmonella from poultry farms in the towns of Bishoftu and Adama and to determine the antimicrobial susceptibility frequency of the isolates. A total of 384 samples were tested for the presence of Salmonella, including 259 feces, 56 eggs, and 69 types of meat, using the ISO, 2002 standard procedures. The raw data were organized, coded, and entered into an Excel spreadsheet before being analyzed with STATA via descriptive analysis with chi-square. From 384 collected samples, 62 (16.15%) isolates were obtained, with 9.9%, 3.65, and 2.6% found in feces, eggs, and meat, respectively. Statistically, there was a significant difference between breeds (*p* value = 0.036). Bovines had the highest prevalence (32.83%), while Saso had the lowest (30.81%). The variation within each sample type, housing condition, and age group was not statistically significant (*p* value >0.05). Antimicrobial resistance was found in 29 (96.77%) of the isolates. Ampicillin and sulphamethoxazole-trimethoprim were effective against all isolates. Salmonella was isolated from various locations, sample types, ages, and breeds, indicating a wider distribution. Salmonellosis detection isolates suggested that it could be an emerging poultry and public health issue. As a result, future research should concentrate on isolating and identifying salmonella from poultry in backyard systems and comparing it to that of an intensive farm, as well as molecular characterization for serotyping and genetic studies, as well as genes responsible for salmonella pathogenicity and drug resistance.

## 1. Introduction

Foodborne pathogens are a major source of economic and health problems around the world [1]. Salmonella is one of the leading causes of foodborne illness worldwide, with 3.7-billion-dollar annual economic loss [2]. It is the leading cause of acute gastroenteritis in several countries and continues to be a major public health concern globally, particularly in developing countries [3]. Although diseases caused by this pathogen have been linked to a wide range of food sources, poultry in particular has been identified as the single most common source of human salmonellosis [4].

Animal-derived foods, particularly poultry and poultry products, are frequently implicated in sporadic cases and outbreaks of human salmonellosis [5]. Poultry and poultry products are a common source of foodborne illness and consistently rank among the top animal sources of Salmonella that enter the human food supply. He also mentioned that humans are exposed to this problem when they consume raw or undercooked food, particularly poultry and egg products [6].

Salmonella is a type of bacteria that is heterogeneous, short bacilli, 0.7–1.5 × 2.5 m, Gram-negative, aerobic or facultative anaerobic, oxidase negative, catalase positive, indole and Voges Proskauer (VP) negative, methyl red and Simmons citrate positive, H_2_S producing, and urea negative. Except for *Salmonella pullorum* and *Salmonella gallinarum*, which are nonmotile, they ferment sugars with gas production, are nonsporogenic, and typically have peritrichous flagella [7]. Around 7.0 is the ideal pH for multiplication; pH values of 9.0 or lower are bactericidal. The ideal temperature range is between 35 and 37°C, with a minimum of 5°C and a maximum of 47°C. Salmonella cannot survive concentrations of salt higher than 9%. It shares a close relationship with the genus *Escherichia* and can be found on every continent in both warm-blooded and cold-blooded animals as well as in nonliving environments [8].

Except for Salmonella serotype *Typhi*, which only produces acid, the majority of salmonellae catabolize a variety of carbohydrates including glucose, mannitol, and maltose into acid and gas. In contrast, lactose, sucrose, and saline are not fermented by the majority of salmonellae [9]. Salmonella falls under the domain of bacteria. Proteobacteria as a phylum, Gammaproteobacteria as a class, enterobacteriales as an order, Enterobacteriaceae as a family, and Salmonella as a genus [10].

Currently, the Centers for Disease Control and Prevention (CDC) employs the Salmonella nomenclatural system recommended by the World Health Organization (WHO) Collaborating Center [11]. Based on differences in their 16S rRNA sequence analysis, the genus Salmonella is divided into two species, *Salmonella enterica* and *Salmonella bongori*. *Salmonella enterica* is classified into six subspecies such as *arizonae*, *diarizonae*, *enterica*, *houtenae*, *indica*, and *salamae* [12].

Currently, about 2541 Salmonella serotypes have been identified [12]. These serotypes can be distinguished by the type of somatic (O) and flagellar (H) antigens present. In addition, *Salmonella typhi* and a few other Salmonella serovars, including Salmonella Dublin, have a capsular polysaccharide virulence antigen [13].

Many animal species, particularly chickens, pigeons, and reptiles, are possible reservoirs for this bacterium [14]. Salmonella is typically transmitted among poultry through a fecal-oral route, most often through the consumption of contaminated food or water. Human transmission can occur via a variety of routes. Consumption of contaminated food products (milk, eggs, and meats), direct contact with animals and their environments, and cross-contamination through direct contact of foods with contaminated surfaces such as stainless steel, hanging material, knives, and buckets where milk is collected are all important mechanisms for pathogens to contaminate food products [15]. Salmonella infections range from gastrointestinal infections characterized by inflammation of intestinal epithelia, diarrhea, and vomiting to typhoid fever, a potentially fatal infection whose severity is determined by the host immune status and the pathogenicity of the bacterium [16]. Young and immunocompromised patients are more vulnerable to dangerous complications, which are typically treated with fluoroquinolones and extended-spectrum cephalosporins, which are commonly used in veterinary medicine [17].

Ethiopia has approximately 56.87 million chickens, the majority (95%) of which are kept in low-input low-output village chicken production systems [18]. Salmonella was found in large numbers and was widely distributed in Ethiopia, according to research. The number of Salmonella outbreaks in humans in the country has increased significantly in recent years. Much more is now known about the scope of foodborne illness and how severe it can be, both in terms of acute illness and long-term consequences. Various percentages of Salmonella isolates were found in Ethiopian towns, according to studies. Furthermore, a high proportion of *S. typhi* isolates are resistant to antimicrobial agents [19].

Salmonella serotypes isolated from animal foods have multidrug resistance frequency, according to studies from various countries [20]. Meat and poultry products have also been implicated in the spread of antimicrobial-resistant zoonotic bacterial pathogens [21]. Several studies on the prevalence and antimicrobial resistance of Salmonella in processed poultry, poultry products, and poultry processing plants have been conducted in other countries, including Agada et al. [22], Orji et al. [23] from Nigeria, Kagambega et al. [24] from Burkina Faso, Al-Abadi and Al-Mayah [25] from Iraq, and Khan et al. [26] from Pakistan.

Regardless, there are more published and unpublished papers in Ethiopia on Salmonella from poultry, dairy cattle, abattoirs of large and small ruminants, and other feed items. Abunna et al. [27], Aseffa et al. [28], and unpublished: Tadesse [29]. However, little is known about Salmonella isolation from poultry products in Ethiopia. As a result, increased and long-term surveillance of the most risk factors is required, as is the isolation of Salmonella from poultry and poultry products. Thus, the objective of the current study was to detect salmonella from poultry products like meat, feces, and eggs, and to analyze the effectiveness of antimicrobial resistance for selected drugs in the study area.

## 2. Methods and Materials

### 2.1. Study Area

From December 2021 to May 2021, the cross-sectional study was carried out in Bishoftu and Adama. 45 kilometers southeast of Addis Ababa, the capital city of Ethiopia. Bishoftu had a long rainy season from June to October and a short rainy season from March to May, the region has a bimodal rainfall pattern and is located at an altitude of 1850 meters above sea level. The region experiences 875 mm of annual rainfall and average high and low temperatures of 26 degrees Celsius and 14 degrees Celsius, respectively. Adama is 99 kilometers to the southeast of Addis Ababa. It is situated at an elevation of 1,712 meters above sea level at 8.54°N 39.27°E. It has a yearly average minimum and maximum temperatures of 18 and 32 degrees Celsius, respectively, and receives 600 to 1,150 mm of rainfall on average [30].

### 2.2. Study Animal

The study animals were poultry from the intensive poultry production systems of the private and government farms in Bishoftu and Adama. Each poultry farm received letters requesting their support before sample collection. Unaffected by age, sex, or color, the randomly selected chicken was evaluated. Owners and workers on the farm were provided information regarding them.

### 2.3. Study Design

A cross-sectional study was carried out to isolate Salmonella from poultry farms between December 2021 and May 2021. The sampling was done using a straightforward random sampling method, and each poultry farm was given a different set of sampling days.

### 2.4. Sample Size Determination and Sampling Techniques

The straightforward random sampling technique was used, giving chickens from intensive farms in Bishoftu and Adama an equal chance of being included in the sample. The necessary sample size for this study was meticulously calculated [31]. Since no research has been done in this area before, a sample size of 50% prevalence has been chosen. A 95% confidence interval and a desired absolute precision of 5% were also factors in the sample size calculation.

Thus, the total sample was 384.

### 2.5. Sample Collection

Fecal (259), eggs (56), and meat (69) samples (*n* = 384) from poultry and poultry houses kept in intensive farms in the study areas were collected for the study. The collected samples were delivered using an icebox to the college of veterinary medicine at Addis Ababa University's public health laboratory in Bishoftu. As soon as the samples were collected, it was cultured; otherwise, it was stored at 4 degrees Celsius for a maximum of 24 hours before being cultured. Due to their selectivity and sensitivity, microbiological culture techniques are still regarded as the “gold standard” for diagnosing Salmonella infections. They have long been the main diagnostic method for identifying Salmonella infections [32].

### 2.6. Bacteriological Methodology

#### 2.6.1. Procedure

The method used to isolate the bacteria was based on ISO protocol 6579 : 2002, “Microbiology of Food and Animal Feeding Stuffs, Horizontal method for the detection of Salmonella species” [33]. The testing of suspect food items and animal feces samples discovered through foodborne disease surveillance programs is intended to be guided by this protocol. This protocol is only meant to be applied to animal waste and food samples. According to this theory, Salmonella must be detected in the following four steps: pre-enrichment in nonselective liquid media, enrichment in selective liquid media, and selective plating on selective solid agar. Suspect isolates were found and verified through screening against five biochemical tests [7].

#### 2.6.2. Antimicrobial Resistance

*Antimicrobial Sensitive Test* (*Disk Diffusion or Disk Plate Technique*). It is the most commonly used method to determine qualitative antibiotic sensitivity tests “in vitro.” It is almost a qualitative way of determining antibiotic sensitivity tests based on diffusion. The most common test medium is called Mueller–Hinton Agar (MHA). Small discs containing different antibiotics, or impregnated paper disks, are dropped in different zones of the culture on Muller Hinton Agar medium. Since the agar plate is a nutrient-rich environment in which bacteria can grow, the antibiotic will diffuse in the area surrounding each tablet, and a disc of bacterial lysis will become visible. The area around the disc where there is no growth of bacteria is called the zone of inhibition. The area around the disc where there is no growth of bacteria is called the zone of inhibition [34].

### 2.7. Data Management and Analysis

The study's raw data were coded, organized, and entered into an Excel spreadsheet using Microsoft® Office Excel 2010. Then, using STATA, a descriptive analysis using chi-square statistics was performed on the data. The analyses' findings will be broken down into proportional descriptions. The percentage was calculated based on the number of samples that tested positive for Salmonella out of the total samples tested as well as the ratio of antimicrobial-resistant isolates to positive samples.

## 3. Results

### 3.1. Distribution of Salmonella Isolation

A total of 384 samples from various poultry farms in the study areas were collected, and 62 (16.15%) of the isolates tested positive for salmonella. Salmonella was found in Bishoftu and Adama, respectively, with a total isolation rate of (59.68%) and (40.32%). Between study areas, there were no statistically significant differences (2 = 0.9106, *p* value = 0.340). Out of 208 samples, the Bishoftu farms had a prevalence of 37 (17.79%). Out of 176 samples, 25 (14.20%) were found in Adema farms, which had a lower prevalence. Meat 10/69 (14.49%), feces 38/259 (14.67%), and eggs 14/56 (25%) were all positive (2 = 3.7975, *p* value = 0.150), indicating that there was no statistically significant difference in the distribution of isolates between the various sample types.

As of 30 (12.93%) out of 232 samples from Saso and 32 (21.05%) out of 152 samples from Bovans Brown, there was a statistically significant association between the distributions of isolates across different poultry breeds (2 = 4.3889, *p* value = 0.036). The distribution of isolates among different ages of poultry did not differ significantly, with 34 (15.96%) out of 213 and 28 (16.37%) out of 171 isolated from young and adult birds, respectively (2 = 0.0119, *p* value = 0.913). It was not statistically significant (2 = 4.3889, *p* value = 0.036) that the isolate distribution varied between chicken production states (detailed results were shown in Table 1).

### 3.2. Frequency of Antimicrobial Resistance Distribution

The 30 positive Salmonella isolates were selected and screened for antimicrobial susceptibility tests against five antimicrobials such as sulphamethoxazole-trimethoprim, gentamycin, streptomycin, ampicillin, and ciprofloxacin. 29 (96.77%) were resistant to one or more of the antimicrobials. Only one isolate from fecal samples was sensitive to the entire five selected antimicrobial drugs. All isolates were susceptible to ampicillin and sulphamethoxazole-trimethoprim. Although all isolates were supposedly susceptible to ciprofloxacin, 4 (13.3%) isolates were intermediately susceptible. In addition, all isolates were supposedly susceptible to gentamycin but 5 (16.7%) isolates were intermediately susceptible. 26 (86.7%) salmonella isolates were resistant to streptomycin while only 2 (6.7%) and 2 (6.7%) isolates were sensitive and intermediately sensitive to streptomycin, respectively. In addition, 5 (16.67%) isolates were resistant to two antimicrobial drugs. The rest 19 (65.52%) of the 30 resistant isolates were only resistant to streptomycin. Only 3 (10%) isolates were resistant to three antimicrobial drugs namely gentamycin streptomycin and ciprofloxacin.

## 4. Discussion

The current study evaluated the antibiogram frequency and isolation and identification of salmonella from poultry products. Salmonella was present in the sample collected overall at a rate of 16.14%. The study found that some of the prevalence of Salmonella isolation was consistent with that reported in Ethiopia and other nations. At 15.5% [28] from chicken table eggs by bacteriological methods in Ethiopia, 15.12% [27] from poultry cloacal swabs, fresh feces, litter samples, chicken feed samples, poultry drinking water, and chicken handlers by bacteriological methods in Ethiopia, and 12.5% [23] from chicken handlers in Nigeria.

Higher prevalence than the current findings has also been reported in Ethiopia and other countries, including 41.9% [35] from fecal samples by the bacteriological method in Ethiopia and 35.7% [36] and 55% [24] in Burkina Faso. This discrepancy may be caused by the protocol followed and variations in chicken management practices between nations and chicken farms.

In Ethiopia and other nations with a lower prevalence than that of the current study, similar studies to isolate Salmonella from various poultry products were carried out. A few instances include the isolation of *Salmonella enterica* serovar *Gallinarum* at a rate of 2.6% [37] from local chicken in Tanzania, the isolation of Salmonella at a rate of 2.5% [38] from clean eggs in Ethiopia, the isolation of *Gallinarum* and *S. pullorum* at a rate of 0.8% [39] from cloacal. The use of primary and pre-enrichment media, the use of a large amount of sample, which increased the likelihood of Salmonella recovery from poultry products as described by ISO, and the pooling of samples in the present study, which was based on Wallace et al. [40] and ISO [33].

These differences (higher or lower prevalence) from the current finding could, in general, be attributed to differences in the isolation technique, sample type and quantity, geographic location, bird breeds, and work quality.

All of the Salmonella isolates used in the current study were derived from feces, meat, and egg samples. As fecal 38 (61.29%), egg 14 (22.58%), and meat 10 (16.13%) were positive out of the total sample, there is no statistically significant difference in the distribution of isolates between different sample types [2 = 3.4380, *p* value = 0.179]. The current percentage of fecal isolates (61.29%; 38/62) differed from the results of Orji et al. [23] in Nigeria (12.5%) and Kagambega et al. [24] in Burkina Faso (55%). 10.33% (16/62) of the salmonella isolates in the current study came from meat samples. The current study did not support the findings of Abunna et al. [27]; and Davies and Hinton [41]; which found no salmonella.

Salmonella prevalence was found to be 2.5% in similar studies to isolate Salmonella from clean eggs conducted in Ethiopia [38]. 14 (or 25%) of the 56 egg samples had results that did not agree with those of the current study. The geographic region, sample size, sampling technique, and breeds of poultry from which the eggs were taken could all be contributing factors to the difference.

Sulphamethoxazole-trimethoprim, gentamycin, streptomycin, ampicillin, and ciprofloxacin were the five antimicrobial drugs tested for antimicrobial susceptibility on the thirty chosen Salmonella isolates. Ampicillin and sulphamethoxazole-trimethoprim were effective against all isolates. Although all isolates should have been able to respond to Ciprofloxacin and Gentamycin, only 4 (13.3%) and 5 (16.7%) isolates showed intermediate susceptibility. The outcome was consistent with those reported by [42], Al-Ledeni et al. [43], Maria [44] from America, Tabo et al. [45] in Chad, and Carramiñana et al. [46] from Spain, who all came to the same conclusion that first-line medications like chloramphenicol, gentamycin, and sulfamethoxazole-trimethoprim as well as currently The findings of Adesiji et al. [47], Cardoso et al. [48], Tsegaye et al. [49], Singh et al. [50] from India, and Antunes et al. [51] from Portugal were in disagreement with the sensitivity results of this study, but they differed with resistant patterns in which tested Salmonellae were highly resistant to ciprofloxacin, gentamycin, and sulfamethoxazole Disagreement may result from various isolate strains as well as poor processing quality.

26 salmonella isolates (86.7%) were streptomycin resistant. The results of the current study were consistent with those of studies by Cardoso et al. [48], Agada et al. [22] from Nigeria, Abunna et al. [27] from Ethiopia, and Davies and Hinton [41], which found that the majority of Enterobacteriaceae family members, including Salmonella, are resistant to antibiotics like aminoglycosides like streptomycin and betalactams. Thus, the most prevalent single resistance was streptomycin (86.7%). These could be brought on by the widespread use of streptomycin in conjunction with penicillin (pin strip), as well as the drug's accessibility from all veterinary medications in Ethiopia, affordability from a neighborhood pharmacy, and frequency of use, which increases salmonella exposure to medications that encourage the emergence of resistance.

Only three isolates (10%) were resistant to gentamycin, streptomycin, and ciprofloxacin, three antimicrobial drugs. Furthermore, 5 isolates (16.67%) were resistant to two antimicrobial drugs. The rest only 21 (70%) of the isolates had streptomycin resistance. This finding disagreed with those of studies by Abunna et al. [27]; which found that 19 of 30 (63.33%) resistance isolates were resistant to four to seven different antimicrobials, Payne et al. [52] on broiler farms, where 96% of the isolates were resistant to more than one antimicrobial agent (s), and Singh et al. [53] on resistance isolated from chicken eggs, poultry farms, and markets, which found that two isolates were resistant to out of 27 multiresistant isolates, five were resistant to five different antimicrobials, according to Jahan et al. [54]. This discrepancy might be caused by the drugs' unavailability, high cost, or affordability, which may prevent both humans and animals from being exposed to them. Antimicrobial resistance against drugs such as ciprofloxacin, sulphamethoxazole-trimethoprim, and gentamycin was less common in Ethiopia because these medications were more expensive for veterinary use.

## 5. Conclusion and Recommendations

The current study demonstrated that Salmonella is prevalent overall at a rate of 16.14% in the study area. One of the leading causes of morbidity and mortality in poultry, salmonellosis is still a distressing public health concern on a global scale. The Salmonella strains' genetic makeup enables them to adapt to a variety of environments, including hosts that are both animal and nonanimal. This makes getting rid of the bacteria more challenging. The majority of isolated Salmonella was found to be sensitive to the majority of the tested antibiotics. Most resistant isolates were only susceptible to one or two antimicrobial medications. Streptomycin resistance was present in the majority of salmonella isolates, which may be related to the drug's widespread use in Ethiopia. However, their use for treating typhoid and salmonellosis in humans and animals should continue with justification, and treatment is necessary if illness persists, especially in immune-compromised people (HIV/AIDS). The recommendations that were made were as follows in light of the aforementioned result and conclusion:

- The public health sectors should create awareness through various pieces of training, workshops, and seminars to inform health stakeholders
- People should be made aware of the need to refrain from practicing serving raw eggs to children or using them as traditional medicine
- Further studies should concentrate on molecular characterization for serotyping and genetic studies and genes responsible for Salmonella's pathogenicity and drug resistance

## Figures and Tables

**Table 1:** Distribution of Salmonella isolates from different locations, breeds, ages, and housing conditions.

Variable	Positive	Total	Prevalence (%)	Χ^2^	*p* value
Study areas					
Bishoftu	37	208	17.79	0.9106	0.340
Adama	25	176	14.20		
Sample type					
Egg	14	56	25.00	3.4380	0.179
Fecal	38	259	14.67		
Meat	10	69	14.49		
Breed					
Saso	30	232	12.93	4.3889	0.036
Bovans Brown	32	152	21.05		
Age					
Young	34	213	15.96	0.0119	0.913
Adult	28	171	16.37		
Housing condition					
Battery cage 18 system		129	13.95	0.7035	0.402
Deep litter 44 system		135	17.25		

## Data Availability

All the datasets generated or analyzed during this study are included in this manuscript.
